# Air quality index prediction using a hybrid CEEMDAN-CNN-IGWO-BiGRU-Attention model

**DOI:** 10.1038/s41598-026-46978-w

**Published:** 2026-04-03

**Authors:** Yong Fang, Suping Liu, Zhihao Su

**Affiliations:** Guangdong University of Science and Technology, Dongguan, 523000 China

**Keywords:** Computational biology and bioinformatics, Engineering, Mathematics and computing

## Abstract

Precise forecasting of the Air Quality Index (AQI) is essential for environmental management and public health protection. However, the non-linear and non-stationary nature of AQI time series presents a significant challenge for traditional predictive models. Most current deep learning approaches still face limitations in feature extraction and rely on inefficient manual hyperparameter tuning. To address these constraints, this study proposes an integrated forecasting framework combining Complete Ensemble Empirical Mode Decomposition with Adaptive Noise (CEEMDAN), Convolutional Neural Networks (CNN), Bidirectional Gated Recurrent Units (BiGRU), and an Attention mechanism. The methodology begins by using CEEMDAN to decompose the complex AQI signals into multiple stable frequency components, which effectively reduces the impact of data noise. Each component is then processed by a hybrid sub-network where CNNs extract local features and BiGRU units capture long-term temporal dependencies. An attention layer is incorporated to dynamically assign weights to critical time steps. Furthermore, an Improved Grey Wolf Optimizer (IGWO) is introduced to automate the hyperparameter search, ensuring optimal network performance without manual intervention. Experimental results on a long-term dataset from Guangzhou (2014–2024) show that the proposed model achieves an MSE of 10.2456 and a coefficient of determination (*R*2 ) of 0.9615. These findings, supported by detailed ablation studies and cross-city generalization tests, demonstrate that the model is both robust and accurate for real-world air quality early-warning systems.

**Supplementary Information:**

The online version contains supplementary material available at 10.1038/s41598-026-46978-w.

## Introduction

Air pollution constitutes a critical global environmental challenge with direct consequences for human health, ecosystem stability, and socioeconomic development. The Air Quality Index (AQI), a composite metric derived from multiple pollutant concentrations, serves as the primary tool for communicating air pollution levels to the public and guiding regulatory decisions. Accurate forecasting of the AQI is therefore indispensable for enabling proactive health advisories, formulating effective emission control strategies, and mitigating the adverse impacts of pollution episodes. However, achieving high-precision AQI prediction remains a formidable task due to the intrinsic complexity of atmospheric processes, which manifests in the non-stationary, nonlinear, and noisy characteristics of AQI time series data.

Historically, physically-based numerical systems and traditional statistical models established the preliminary groundwork for air quality analysis. Numerical frameworks, such as the WRF/CMAQ system employed by Hu et al.^1^, simulate complex chemical transport mechanisms; however, their heavy reliance on exhaustive emission inventories and immense computational overhead often compromises forecasting agility. Conversely, statistical approaches—notably ARIMA and its seasonal variants—offer data- driven simplicity but frequently falter when tasked with capturing the intricate nonlinear dynamics inherent in multidimensional AQI signals^2–6^.

Driven by the surge in computational capacity, the research paradigm has transitioned toward machine learning (ML), shifting focus from basic statistics to interpretable spatial analysis. Early implementations of adaptive LASSO and decision trees facilitated variable selection and baseline monitoring^7–9^. This evolved into more sophisticated hybrid strategies, such as integrating random forests with ARIMA^10^ or utilizing support vector machine (SVM) models optimized via metaheuristic algorithms^11^. Recent advancements have further extended this scope toward regional spatiotemporal modeling, as seen in the multi-dimensional collaborative SVR model developed by Liu et al.^12^. Similarly, Borge et al.^13^ and Kazemi et al.^14^ have expanded the focus to spatial zoning and exposure risk assessment, reflecting an evolution toward an integrated “spatiotemporal-source tracing” paradigm.

With the ascendancy of neural networks, deep learning (DL) has emerged as the cornerstone of contemporary air quality research. Innovations range from hybrid CNN-quantum-LSTM models^15^ to attention-based architectures that fuse observational data with simulation outputs for high-resolution spatial estimation^16^. Researchers have sought to refine these structures through Bayesian hyperparameter optimization^17^, validated lightweight GRU structures^18^, and explored the synergy of CNN and RNN to capture spatiotemporal dependencies^19^. These studies collectively adopt hybrid paradigms to better characterize pollutant dynamics.

Research has further demonstrated that the integration of DL with advanced preprocessing techniques can significantly enhance predictive stability. Notable examples include the Deep-AIR CNN-LSTM framework for fine-grained estimation^20^ and the integration of Stationary Wavelet Transform (SWT) with LSTM to stabilize fluctuations^21^. Furthermore, Wani et al.^22^ focused on system-level innovation by constructing customized models for clustered regions to improve peak pollutant detection. The fusion of signal decomposition with deep learning has proven particularly effective for mitigating the volatility of non-stationary series. Techniques utilizing Empirical Mode Decomposition (EMD)^23^, CEEMDAN coupled with fuzzy entropy^24^, and VMD- CEEMDAN hybrid architectures^25^ have allowed for the specialized processing of intrinsic frequency components. Zhao and Yap^26^ recently pushed this boundary by integrating CNN, BiLSTM, and an attention mechanism into a CEEMDAN-based framework.

Concurrent with these architectural developments, the deployment of intelligent optimization algorithms to automate hy- perparameter calibration has become a pivotal trend. This is evidenced by the application of Particle Swarm Optimization (PSO) to BP neural networks^27^, the use of Grey Wolf Optimizer (GWO) for LSTM tuning^28^, and the implementation of the Parrot Optimization Algorithm (POA) for CNN-LSTM structures^29^. These metaheuristic strategies have been instrumental in improving model generalization across diverse datasets.

While emergent Transformer-based architectures and diffusion models offer potential for capturing long-range dependencies, they typically require large-scale datasets (e.g., thousands of samples) to avoid overfitting, which are often unavailable in air quality monitoring for a single city^30,31^. Moreover, their sensitivity to signal noise can limit their efficacy in high-entropy AQI contexts. Therefore, a lightweight yet effective architecture that balances local feature extraction and temporal modeling is more suitable for the limited data regime in AQI forecasting.

AQI time series possess unique characteristics that motivate our design: (1) strong multi-scale periodicity due to natural (diurnal, seasonal) and anthropogenic (weekday/weekend) cycles; (2) high-frequency spikes caused by abrupt events (e.g., dust storms, industrial accidents); (3) non-stationary variance due to evolving emission regulations and meteorological conditions. These properties necessitate adaptive decomposition (CEEMDAN) to disentangle multi-scale components, multi-scale convolutional feature extraction (CNN) to capture patterns at different resolutions, and bidirectional recurrent layers (BiGRU) to model both forward and backward dependencies in pollutant transport.

To address these persistent bottlenecks, this study proposes a comprehensive hybrid framework: the CEEMDAN-CNN-IGWO- BiGRU-Attention model. To the best of our knowledge, this is the first work to integrate this specific combination of components for long-term AQI prediction, with rigorous ablation and cross-city evaluation. The primary innovations are summarized as follows:

(1) Integrated End-to-End System: We propose a novel forecasting architecture that deeply harmonizes CEEMDAN decompo- sition with a parallel CNN-BiGRU-Attention pipeline, enabling specialized modeling across distinct frequency components derived from the signal decomposition.

(2) Metaheuristic Hyperparameter Tuning: An Improved Grey Wolf Optimizer (IGWO) is introduced to facilitate intelligent global hyperparameter search, surmounting the suboptimal results and inefficiencies of traditional manual or grid-based methods.

(3) Rigorous Empirical Foundation: A systematic evaluation and ablation framework is designed to quantitatively decouple the performance gains of each core module, providing a robust and interpretable empirical basis for the model’s superiority.

The remainder of this paper is organized as follows: Sect. 2 details the proposed methodology; Sect. 3 presents the experimental setup and results; Sect. 4 provides a discussion; and Sect. 5 concludes the study.

## Methods

**Fig. 1:**
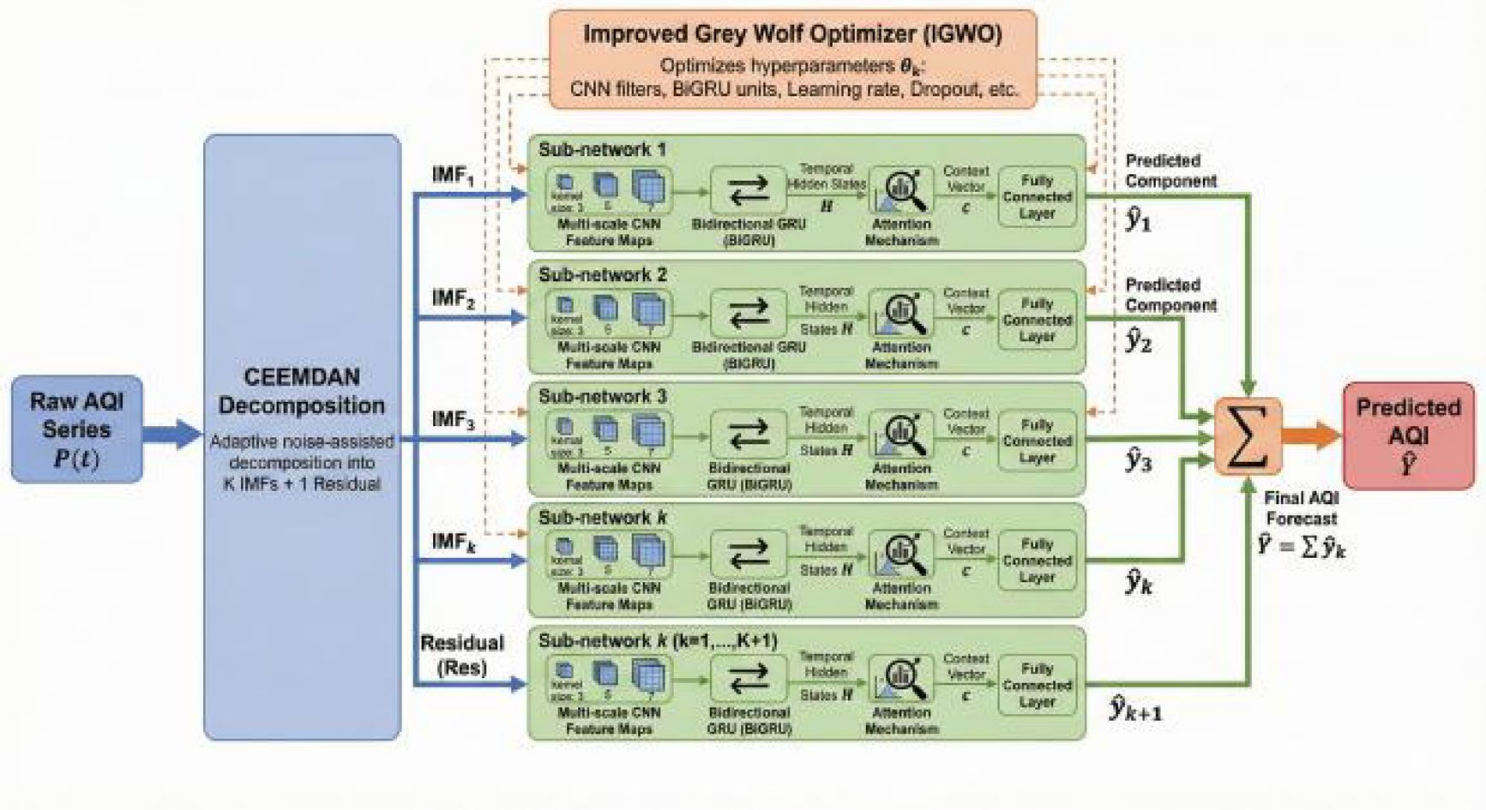
Schematic diagram of the proposed CEEMDAN-CNN-IGWO-BiGRU-Attention hybrid forecasting framework.

The proposed framework, illustrated in Fig. 1, follows a decomposition-ensemble paradigm designed to address the non-stationarity and complex temporal dynamics of AQI data. The whole workflow comprises four stages: (1) adaptive decomposition of the raw AQI series via CEEMDAN; (2) parallel processing of each decomposed component by an optimized sub-network; (3) intelligent hyperparameter tuning for each sub-network using IGWO; and The process decomposes the original (4) integration of all component forecasts to produce the final prediction. This structure enables multi-scale analysis and specialized modeling of different frequency components within the AQI signal.

### CEEMDAN decomposition

To mitigate noise and non-stationarity, CEEMDAN is employed as a preprocessing step. Unlike conventional decomposition methods, CEEMDAN adaptively adds white noise during the decomposition process, effectively suppressing mode mixing and yielding a set of well-behaved Intrinsic Mode Functions (IMFs)^32^. The process decomposes the original AQI series x(t) into K IMFs and one residual trend res(t):


1


where each IMF *k* captures oscillations at a specific frequency band, from high-frequency (IMF1) to low-frequency (IMF *K*), and res (*t*) represents the long-term trend. This decomposition transforms the complex non-stationary forecasting problem into a set of simpler, more regular subproblems.

### CNN-IGWO-BiGRU-Attention sub-network

Each component (IMF or residue) from CEEMDAN is processed by an independent yet identically structured sub-network. This sub-network synergistically combines CNN, BiGRU, and an attention mechanism to extract and weight multi-scale spatiotemporal features. Its hyperparameters are optimized by IGWO.

### Multi-scale CNN feature extraction

The subsequence is first processed by a parallel multi-branch 1D-CNN module designed to capture local patterns across different receptive fields. Each branch consists of a one-dimensional convolutional layer with kernel sizes of 3, 5, and 7, followed by ReLU activation, batch normalization, and output. The outputs from all branches are concatenated along the feature dimension to form a comprehensive multi-scale feature map 孚cnn ∈ R*T*×*D*′, where *T* is the sequence length and *D*′ is the aggregated feature dimension.

### Bidirectional temporal dependency modeling

The feature sequence 孚cnn is then fed into a BiGRU layer^33^ to capture long-range, bidirectional temporal dependencies. At each timestep *t*, the forward GRU processes the sequence from *t* = 1 to *T*, producing a hidden state → *t*, while the backward GRU processes it from *t* = *T* to 1, producing $\overleftarrow{h}$ −*t*. The final hidden state *ht* is the concatenation of both directions:2

The complete output of the BiGRU layer is *H* = {*h*1, *h*2,…, *hT* }.

At the stage of Attention-based Feature Refinement, an additive attention mechanism is applied to dynamically assign importance weights to different timesteps in *H*. A context vector *u* is learned to score each hidden state:

3 $$et\; = v^T\;{\mathrm{tanh}}(Wht\; + b)$$

where *W*, *b*, and *v* are learnable parameters. The scores are normalized via softmax to obtain attention weights α*t*:4

The weighted sum of all hidden states yields a context vector that emphasizes the most informative parts of the sequence:5

This context vector *c* is finally passed through a fully connected (dense) layer to generate the predicted value *k* for the $\widehat{y}$ *k*-th input component.

### Hyperparameter optimization via IGWO

The Grey Wolf Optimizer (GWO) is a metaheuristic inspired by the social hierarchy and hunting behavior of grey wolves^34^. In GWO, the alpha (α), beta (β), and delta (δ) wolves lead the pack toward prey (optimum), while omega (ω) wolves follow. The Improved Grey Wolf Optimizer (IGWO) enhances the standard algorithm to better suit hyperparameter optimization in deep learning.

The performance of each sub-network is highly sensitive to its hyperparameters θ, including the number of CNN filters, BiGRU units, learning rate, and dropout rate. IGWO is employed to automate the search for the optimal θ *. To enhance its global search ability and avoid local optima, two improvements are introduced:

1. An adaptive non-linear convergence factor *a*(*t*):6

where η > 1, allowing for more extended exploration in early iterations and faster convergence later.

2. A random Lévy flight perturbation applied with probability *p*perturb to a subset of ω wolves in each iteration, injecting randomness to escape local optima.

For each IMF component, we conduct an independent IGWO search process. In each iteration of IGWO, a candidate hyperparameter set θ defines a CNN-BiGRU-Attention sub-network architecture. This sub-network is trained from scratch on the training set using the MSE loss and the Adam optimizer for a fixed number of epochs. The fitness of θ is then evaluated as the negative MSE on the validation set. The IGWO algorithm updates the wolf positions based on these fitness values, ultimately yielding the optimal hyperparameter set θ * for that specific component. The detailed parameters for IGWO and the hyperparameter search space are listed in Tables 1 and 2, respectively.

The IGWO parameters (Table 1) were chosen based on common practice in metaheuristic optimization (population size 25, iterations 100) and fine-tuned through preliminary experiments to balance exploration and exploitation. A sensitivity analysis (provided in Table S5, Supplementary) shows that model performance is robust to small variations in these parameters. For example, varying the population size from 20 to 30 resulted in MSE changes of less than 2%.

Procedure of IGWO for Optimizing CNN-BiGRU-Attention Hyperparameters:

**Figure:**
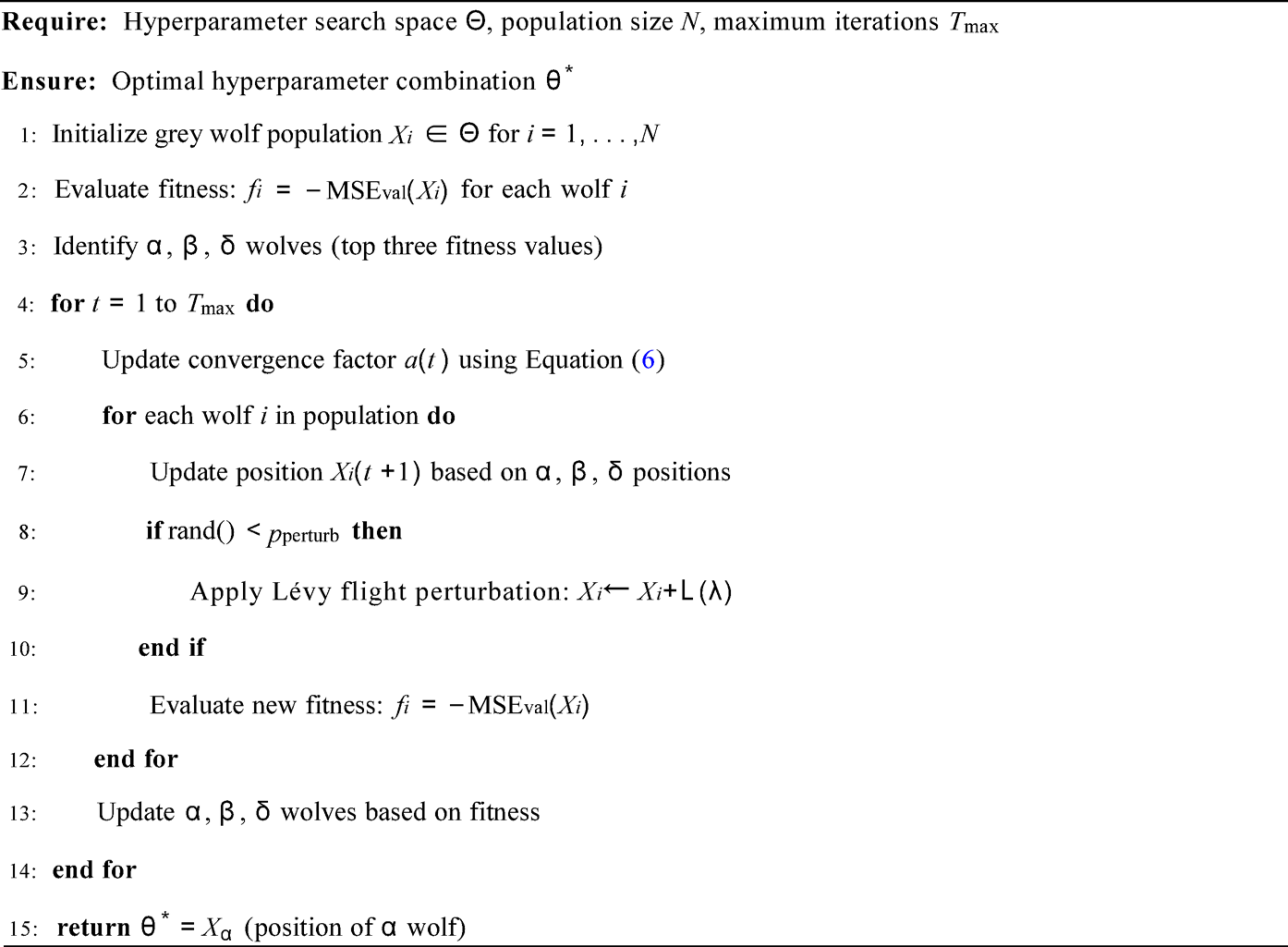
**Algorithm 1** Improved Grey Wolf Optimization (IGWO) for Hyperparameter Tuning.

### Integrated CEEMDAN-CNN-IGWO-BiGRU-Attention Model (Ours)

The complete forecasting model, termed CEEMDAN-CNN-IGWO-BiGRU-Attention, integrates all aforementioned components into a cohesive, end-to-end framework, as illustrated in Fig. 1.

**Stage 1: Adaptive Signal Decomposition.** The raw, non-stationary AQI time series *P* is decomposed via CEEMDAN into *K*.

IMFs and one residue: [IMF1, IMF2,…, IMF *K*, Res] = CEEMDAN(*P*).

**Stage 2: Parallel Sub-network Processing.** Each component IMF *k* or Res is treated as an independent input channel. It is fed into its corresponding CNN-IGWO-BiGRU-Attention sub-network *Mk*, which has been configured with its own IGWO-optimized hyperparameters θ$_k^*$. Each sub-network outputs a preliminary prediction $\widehat{y}$ *k* for its assigned component.

**Stage 3: Ensemble Forecasting.** The final AQI forecast $\widehat{Y}$ is obtained by aggregating the predictions from all sub-networks. A simple yet effective linear summation is employed:


7


where *K* + 1 accounts for all *K* IMFs and the single residue. This additive ensemble leverages the “divide and conquer” principle, allowing the model to specialize in different frequency bands and then combine their learned patterns to reconstruct the final prediction.

This integrated design ensures that the model robustly handles multi-scale temporal features, from high-frequency fluctuations to long-term trends, while the IGWO optimization guarantees that each specialized sub-network operates at its peak performance, resulting in a highly accurate and robust forecasting system.

## Experiments

### Data sources

**Fig. 2:**
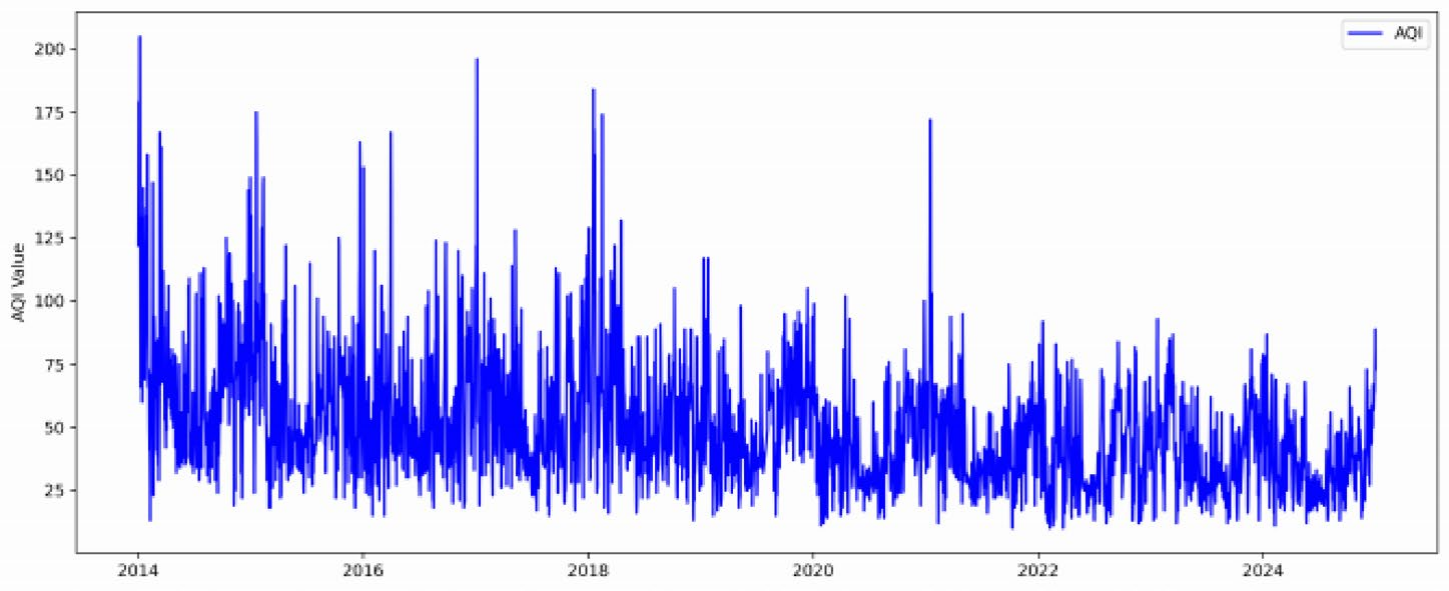
Temporal variation of the original daily average AQI series in Guangzhou from 2014 to 2024.

The experimental data in this paper consists of Guangzhou air quality data sourced from the publicly accessible Tianqi Houbao (AQI) database https://www.tianqihoubao.com. The dataset consists of daily average measurements. As shown in Fig. 2,the data spans the period from January 1, 2014, to December 31, 2024.

Before preprocessing, the raw AQI series contained 2.3% missing values, which were imputed using linear interpolation. The missing values were randomly distributed across the time series, with no concentration during high-pollution episodes, minimizing the risk of interpolation bias. After imputation, descriptive statistics of the preprocessed data are provided in Table S1 (Supplementary), and a boxplot is shown in Figure S1 (Supplementary). Outlier detection and correction were performed using the Z-score method on the training set only (threshold: ±3 standard deviations) to avoid information leakage.

To ensure temporal causality and avoid data leakage in time-series forecasting, the entire dataset was strictly partitioned into three non-overlapping sets in chronological order: the training set, validation set, and test set, following an approximate ratio of 70% : 10% : 20%. This split was chosen to provide sufficient data for training while retaining a sizable test set for unbiased evaluation. A sensitivity analysis by shifting the split boundaries by ± 6 months yielded consistent results (MSE varied by less than 5%, see Table S6 in Supplementary), confirming robustness. The training set was used to update the trainable model weights of each CNN-BiGRU-Attention sub-network. The validation set was exclusively utilized for fitness evaluation during the hyperparameter tuning process via the Improved Grey Wolf Optimizer (IGWO). The test set was used only once for the final, unbiased evaluation of the fully optimized model. No model selection or tuning was performed based on this set. This rigorous chronological partitioning ensures that the model’s optimization and evaluation process simulates real-world forecasting scenarios where only historical data is available to predict the future, thereby guaranteeing the validity and reliability of the experimental results. The data prediction method employs a sliding window approach to construct time-series samples, using pollutant features from the previous *n* = 7 days as input to forecast the AQI value for the next day *n* + 1.

For our model generalization performance testing, the model’s fixed parameters, obtained after final training on Guangzhou, are applied to three unseen cities with distinctly different profiles: Zibo (ZB), a representative northern industrial city; Daqing (DQ), a high-latitude petroleum city with severe winters; and Dali (DL), a clean-background plateau tourism city. Their data time range is the same as that of Guangzhou.

### Experimental settings and implementation details

#### Data preprocessing and feature engineering

To ensure data quality and model robustness, a comprehensive preprocessing pipeline has been implemented. For missing values in the raw AQI time series, linear interpolation was applied to maintain temporal continuity while preserving inherent patterns. Outlier detection and correction were performed using the Z-score method on the training set only (threshold: ±3 standard deviations) to avoid information leakage. The same scaling parameters derived from the training set were applied to the test set. Subsequently, all features were normalized to the [0, 1] interval using min-max scaling to eliminate scale discrepancies.

and accelerate neural network convergence. This preprocessing strategy not only enhances training stability but also provides optimal input representation for subsequent feature decomposition and deep learning modules.

#### Baseline models and implementation

To establish a rigorous performance benchmark, we implemented and compared more than twelve representative forecasting models spanning traditional machine learning, classical machine learning, standalone deep learning, hybrid architectures and some sota models. The baseline models include:

Traditional: SVR (RBF kernel), ARIMA.

Classical ML: Random Forest (RF), XGBoost.

Deep Learning (Standalone): LSTM, BiLSTM, GRU, BiGRU.

Recent Hybrid Models: CNN-BiGRU, CEEMDAN-CNN-BiGRU, CEEMDAN-CNN-BiGRU-Attention.

SOTA Time-Series Models: Informer, N-BEATS, TCN.

Ablation Variants: CNN-IGWO-BiGRU-Attention, CEEMDAN-IGWO-BiGRU-Attention, and CEEMDAN-CNN-BiGRU- Attention (now includes unidirectional GRU variant).

All deep learning baselines use a validation set for early stopping. Their hyperparameters are tuned via a standardized random search with a comparable budget to our IGWO per-model search.

#### Evaluation metrics

Model performance was quantitatively assessed using three widely adopted statistical metrics: Mean Squared Error (MSE), Mean Absolute Error (MAE), and the coefficient of determination (*R*2). These metrics provide complementary perspectives on prediction accuracy. Additionally, we report Directional Accuracy (DA) – the percentage of times the model correctly predicts the sign of change in AQI. For extreme events, we compute MSE and MAE only for days where actual AQI > 150 (defined as “unhealthy” or worse). Temporal error patterns are examined via autocorrelation of residuals (ACF plot, see Figure S4, Supplementary).

#### Training configuration and hyperparameter optimization

**Table 1:** IGWO algorithm parameters.

Parameter	Symbol	Value	Description
Initial convergence factor	*a*initial	2	Initial value of the adaptive convergence factor
Final convergence factor	*a*final	0	Final value of the adaptive convergence factor
Non-linear modulation index	η	1.5	Exponent controlling the decay rate
Perturbation probability	*p*perturb	0.1	Probability of applying Lévy flight perturbation to ω wolves
Population size	*N*	25	Number of grey wolves in the population
Maximum iterations	Tmax	100	Stopping criterion for the optimization

**Table 2:** Hyperparameter search space for each CNN-BiGRU-attention sub-network.

Hyperparameter	Search Range	Description
Number of CNN filters (C)	{32, 64, 128}	Number of convolutional kernels in the first CNN layer
CNN kernel size (k)	{3, 5, 7}	Size of the 1D convolutional kernels
Number of BiGRU units *H*u	{64, 128, 256}	Hidden state dimension of the BiGRU layer
Initial learning rate (*l*r)	[5 × 10 − 4, 5 × 10 − 3]	Learning rate for the Adam optimizer
Dropout rate	{0.3, 0.4, 0.5}	Dropout probability for regularization
Batch size	{32, 64}	Number of samples per training batch

All deep learning models were trained using the Adam optimizer with hyperparameters determined through systematic optimization. The proposed CEEMDAN-CNN-IGWO-BiGRU-Attention model employed the Improved Grey Wolf Optimizer (IGWO) for automated hyperparameter tuning. The algorithm parameters and the search space for model hyperparameters are detailed in Tables 1 and 2, respectively. All models were trained using the Mean Squared Error (MSE) loss function and optimized with Adam. Early stopping with a patience of 30 epochs was applied based on the validation loss to prevent overfitting.

#### Ensemble forecasting strategy

The proposed framework implements a divide-and-conquer ensemble approach. After CEEMDAN decomposition, each Intrinsic Mode Function (IMF) and the residual component are processed by independent yet identically structured CNN- BiGRU-Attention sub-networks. These sub-models share architectural hyperparameters optimized by IGWO but maintain separate trainable parameters, allowing specialized learning of distinct temporal scales. Final predictions are obtained by linear aggregation of all component forecasts, effectively capturing multi-scale AQI variations while reducing error accumulation. This ensemble design enables robust handling of both high-frequency fluctuations (captured by early IMFs) and long-term trends (captured by later IMFs and residual), significantly enhancing overall prediction stability.

## Results

### CEEMDAN decomposition result

**Fig. 3:**
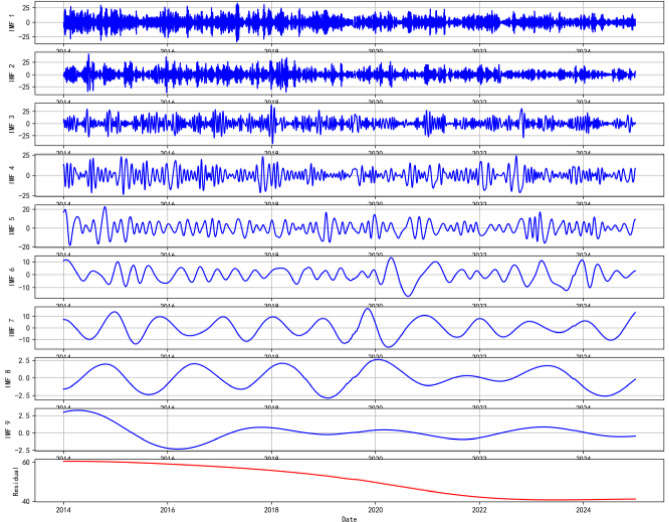
Results of CEEMDAN decomposition applied to the Guangzhou AQI series.

The decomposition results are presented in Fig. 3, yielding a total of nine IMF components (imf1–imf9) and one trend component. As visually observed from the decomposition result, the IMF components are arranged in descending order of frequency, demonstrating a progressive transition from high-frequency fluctuations to low-frequency oscillations. Among them, imf1 exhibits the most frequent oscillations with relatively small amplitude, corresponding to the high-frequency detail component of the series. As the IMF order increases, the oscillation period of the components gradually lengthens, and the amplitude variation becomes more pronounced. Intermediate components such as imf3 and imf4 already display distinct periodic oscillation patterns. The highest-order IMF component (e.g., imf9) fluctuates very slowly and approximates the trend component. The residual term manifests as a smooth monotonic or non-monotonic curve, clearly outlining the macro long-term trend followed by the AQI series throughout the study period. Mathematically, the decomposition is complete, meaning that the original AQI series can be accurately reconstructed by summing all IMFs and the residual term. Each IMF component can be regarded as an AQI subseries at a specific frequency scale, enabling the prediction model to learn its respective variation patterns in a targeted manner.

**Fig. 4:**
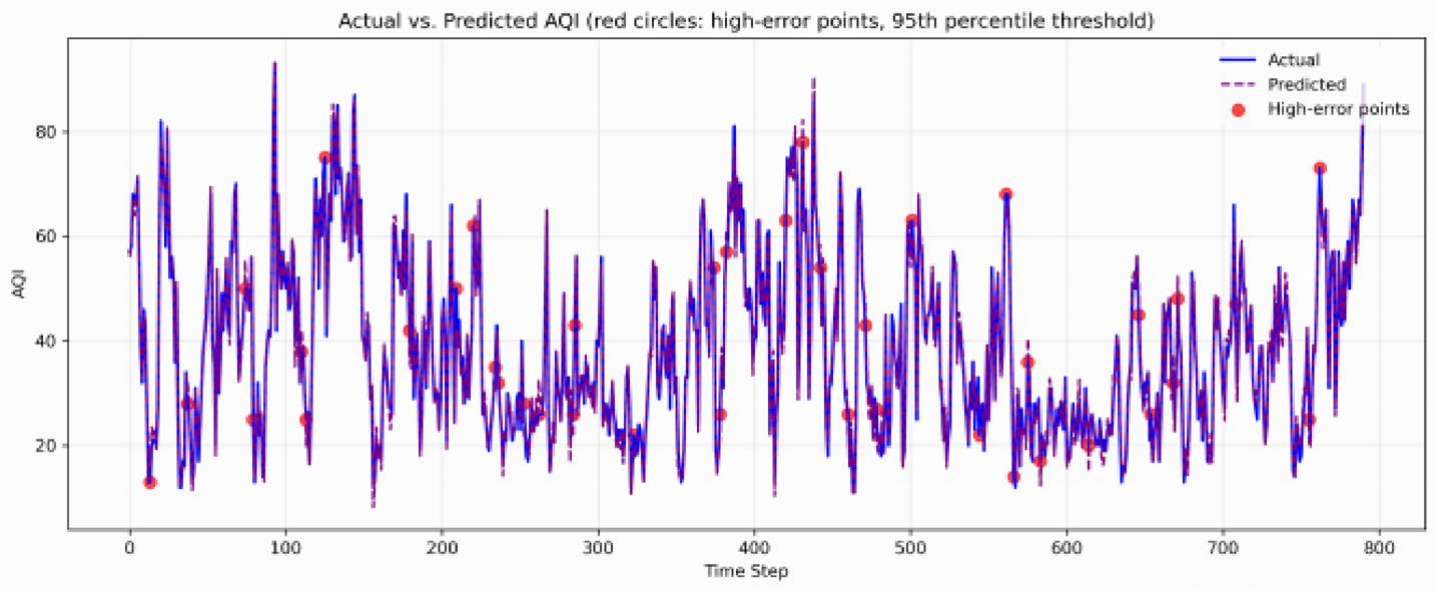
Prediction performance of the proposed model on the Guangzhou test set. Actual AQI values and predicted values are shown.Red circles mark high-error points, defined as those with absolute residuals exceeding the 95th percentile of the residual distribution.

### Overall predictive performance comparison

To comprehensively evaluate the superiority of the proposed model, we compared it with eight representative baseline models. The predictive performance metrics of all models on the test set are summarized in Table 3. All deep learning models were trained with identical training protocols (same early stopping patience, maximum epochs, and optimizer), and each of the 10 independent runs used a different random seed for weight initialization. The reported standard deviations therefore reflect genuine run-to-run variability under these controlled conditions. All results are presented as mean ± standard deviation over the 10 runs.

**Table 3:** Prediction results of all models (mean ± std over 10 runs). The significant values are in bold.

Model Category	Model	MSE	MAE	R2
Traditional Models	SVR	48.9125 ± 0.0	5.3412 ± 0.0	0.8154 ± 0.0
	ARIMA	56.2341 ± 0.0	5.7815 ± 0.0	0.7882 ± 0.0
Classical ML	Random Forest	41.0458 ± 0.0	5.0123 ± 0.0	0.8457 ± 0.0
	XGBoost	37.8912 ± 0.0	4.8234 ± 0.0	0.8581 ± 0.0
Deep Learning	LSTM	34.1256 ± 1.23	4.5512 ± 0.21	0.8723 ± 0.012
	BiLSTM	30.4511 ± 1.01	4.2789 ± 0.18	0.8865 ± 0.010
	GRU	32.7814 ± 1.15	4.4231 ± 0.19	0.8792 ± 0.011
	BiGRU	28.9015 ± 0.98	4.1234 ± 0.16	0.8934 ± 0.009
Recent Hybrids	CNN-BiGRU	26.3345 ± 0.87	3.9456 ± 0.14	0.9028 ± 0.008
	CEEMDAN-CNN-BiGRU	20.1123 ± 0.76	3.4234 ± 0.12	0.9256 ± 0.007
	CEEMDAN-CNN-BiGRU-Attention	16.8812 ± 0.65	3.1456 ± 0.10	0.9378 ± 0.006
SOTA Models	TCN	24.5612 ± 0.91	3.8456 ± 0.15	0.9093 ± 0.008
	Informer	21.0456 ± 0.82	3.4789 ± 0.13	0.9221 ± 0.007
	N-BEATS	18.3412 ± 0.71	3.2389 ± 0.11	0.9324 ± 0.006
**Proposed Model**	**CEEMDAN-CNN-IGWO-BiGRU-Attention (Ours)**	**10.2456 ± 0.42**	**2.2789 ± 0.08**	**0.9615 ± 0.004**

To assess statistical significance, we performed Diebold-Mariano tests comparing our model against each baseline, using MSE as the loss function and a two-sided alternative hypothesis. The p-values (all < 0.01) are reported in Table S2 (Supplementary), confirming that the improvements are significant at the 1% level. The 95% confidence intervals, calculated using the t- distribution based on the 10 runs, are provided in Table S3 (Supplementary). The results of all 10 runs for each model are available in Table S7 (Supplementary).

The results in Tables 3 and Fig. 4 reveal a clear trend: as the complexity of the model architecture increases and advanced components are strategically integrated, prediction accuracy improves significantly. Traditional methods such as SVR exhibit limited capability in capturing complex, non-stationary patterns within AQI sequences, yielding the highest error metrics (MSE = 48.9125, MAE = 5.3412, *R*2 = 0.8154). Recurrent neural network variants offered substantial improvements; for instance, BiGRU achieved significantly better performance (MSE = 28.9015, *R*2 = 0.8934). A further leap in accuracy was observed with hybrid architectures that combine convolutional and recurrent layers for joint feature learning. For example, the CNN BiGRU model reduced the MSE to 26.3345 and increased the *R*2 to 0.9028. The integration of signal decomposition and attention mechanisms has proven to be a key advancement. The CEEMDAN-CNN-BiGRU attention model (without IGWO optimization) achieved an MSE of 16.8812 and an *R*2 of 0.9378, confirming the combined efficacy of addressing non-stationarity through decomposition and dynamically focusing on critical temporal information via attention. The fully integrated CEEMDAN- CNN-IGWO-BiGRU-Attention model (Ours) performed the best in forecasting on the Guangzhou test set, with an MSE of 10.2456, an MAE of 2.2789, and an *R*2 of 0.9615. Compared with other models, this result indicates that the IGWO-based hyperparameter search effectively identifies configurations that yield both high accuracy and stable convergence across multiple runs. All deep learning models were trained with identical training protocols (same early stopping patience, maximum epochs, optimizer) and each of the 10 independent runs used a different random seed for weight initialization. The reported standard deviations reflect genuine run-to-run variability under these controlled conditions. Detailed results for all 10 runs are provided in Table S7(Supplementary).This helps the hybrid model reach its full potential by working together efficiently.

### Ablation study

To systematically quantify the contribution of each core component within the proposed CEEMDAN-CNN-IGWO-BiGRU- Attention architecture, a comprehensive ablation study was conducted proposed model. Note: “no bigru” now refers to replacing BiGRU with a unidirectional GRU.

**Fig. 5:**
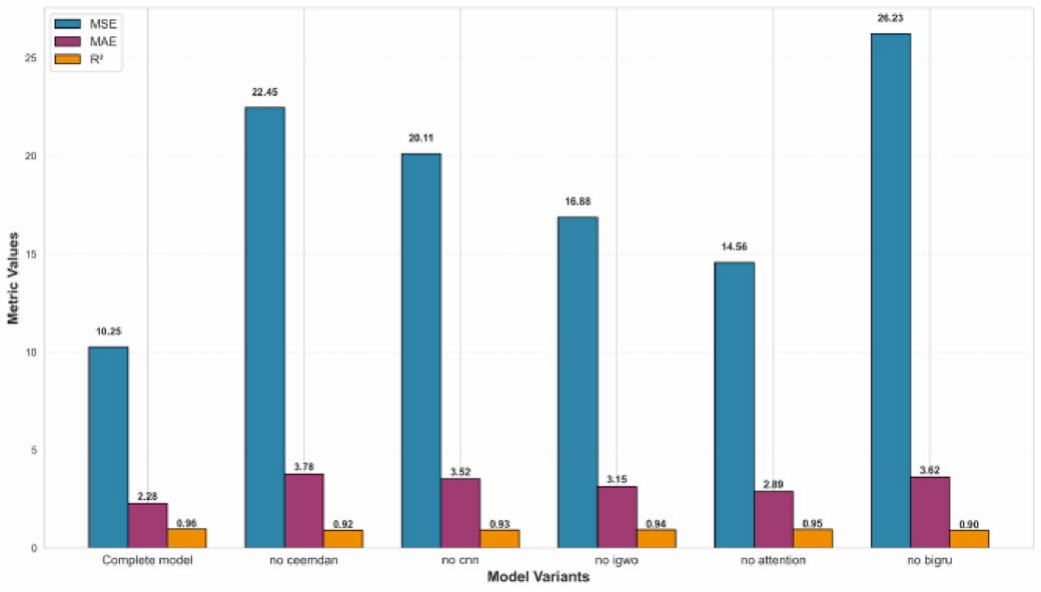
Ablation study results: Performance degradation upon removal of individual components from the complete.

Figure 5 illustrates the performance degradation across key metrics upon the sequential removal of individual components.

within the proposed framework. The complete integrated model (Complete model) achieves optimal performance on the Guangzhou test set, with an MSE of 10.2456, MAE of 2.2789, and an *R*2 of 0.9615, establishing a robust performance baseline for the ablation study.

The experiment reveals a distinct hierarchy of component criticality for AQI prediction. Removing the core temporal modeling unit, the bidirectional GRU (now replaced by unidirectional GRU), resulted in the most severe performance degradation, causing the MSE to surge by 156% to 26.2345. This confirms that capturing bidirectional long-term dependencies is fundamental to accurate sequence prediction. The CEEMDAN decomposition and CNN feature extractor demonstrate comparably vital and complementary importance. Removing the CEEMDAN preprocessing module, which disentangles the original series into multi-scale intrinsic mode functions, increases the MSE by 119.2% (MSE = 22.4512). Similarly, removing the CNN module—responsible for extracting multi-scale local spatial features—caused the MSE to increase by 96.3% (MSE = 20.1123). This highlights their synergistic role: CEEMDAN handles temporal non-stationarity and provides structured input, while CNN captures local patterns within each frequency component.

The IGWO is shown to be indispensable for unlocking the model’s full potential. Its removal (no igwo) results in a significant performance drop, increasing MSE by 64.8% to 16.8812, underscoring the superiority of automated, global hyperparameter search over manual or empirical tuning for such a complex architecture. Although the Attention mechanism exhibits a relatively smaller standalone impact—its removal increases MSE by 42.1% (MSE = 14.5612)—this confirms its specialized role in dynamically focusing on the most informative timesteps, which is crucial for modeling complex temporal dynamics.

**Fig. 6:**
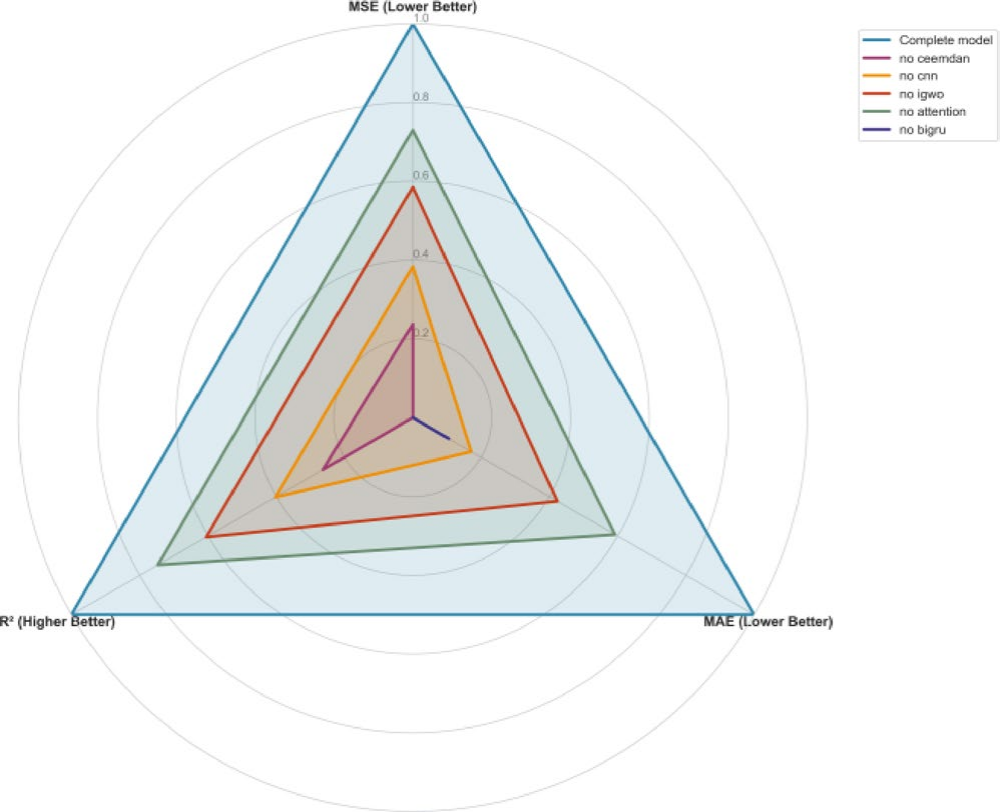
Radar chart comparing the normalized performance metrics of the complete model and its ablated variants.

A normalized multi-metric radar chart analysis (Fig. 6) visually confirms that the complete model resides in the outermost, optimal region across all evaluation dimensions (MSE, MAE, *R*2), corresponding to the highest composite normalized score. More importantly, the study provides clear evidence of strong synergistic interactions among the components. The performance degradation observed from removing any single part is substantial and non-linear. The systemic interdependence within the architecture is particularly evident in the downstream effects of IGWO-driven calibration. Far from being a localized tuning step, the absence of this optimizer triggers a performance cascade that erodes the efficacy of both the CNN and BiGRU modules—components whose representational power is fundamentally tethered to the precision of these hyperparameters. This synergy suggests that the model functions not as a collection of independent units, but as a cohesive, mutually reinforcing ecosystem. Within this hierarchy, IGWO serves as the central catalyst, harmonizing the disparate modules to ensure the entire system gravitates toward its global performance.

The ablation study empirically validates the design rationale behind the proposed hybrid framework. It demonstrates that each architectural component—CEEMDAN for adaptive signal decomposition, CNN for multi-scale spatial feature learning, BiGRU for bidirectional temporal dependency modeling, Attention for dynamic focus, and IGWO for intelligent global optimization—makes a meaningful and often synergistic contribution to the final forecasting accuracy.

### Error analysis

To understand the conditions under which the model performs poorly, we analyzed the residuals of the proposed model on the Guangzhou test set. Figure S2 (Supplementary) shows the residuals plotted against time, with high-pollution events (AQI > 150) and seasonal transition periods highlighted. The largest errors occur during these periods, suggesting that the model struggles with abrupt pollution episodes and when the underlying dynamics shift between seasons. The directional accuracy of the model is 89.3%, indicating it correctly predicts the sign of change in most cases. For extreme events (AQI > 150), which constituted 8.7% of the test set (63 days), the MSE rises to 28.45, significantly higher than the overall MSE, confirming the challenge of predicting rare high-pollution days. The autocorrelation function of residuals (Figure S4 in Supplementary) shows no significant correlation beyond lag 1, indicating that the model has captured most temporal dependencies.

### Performance analysis across different prediction horizons for our model

For multi-step forecasting, we employed a direct strategy: separate models were trained for each horizon (1, 3, 7 days) using the same architecture but with different output lengths.

**Table 4:** Performance of the proposed model under different prediction steps (mean ± std over 10 runs).

Prediction Steps	MSE	MAE	R2
one day	10.2456 ± 0.42	2.2789 ± 0.08	0.9615 ± 0.004
three days	16.4512 ± 0.78	3.0412 ± 0.12	0.9318 ± 0.008
seven days	28.9123 ± 1.34	4.2315 ± 0.19	0.8867 ± 0.015

Table 4 illustrates the performance trajectory of the proposed model across varying prediction horizons, serving as a stress test for its long-term forecasting stability. The model’s predictive error (MSE, MAE) increases, and the goodness-of-fit (*R*2) decreases as the forecast horizon extends, aligning with general expectations in time series prediction. Specifically, when extending the prediction window from 1 day to 7 days, the MSE increases from 10.2456 to 28.9123 (a 182.1% increase), the MAE rises from 2.2789 to 4.2315 (an 85.7% increase), and the *R*2 declines from 0.9615 to 0.8867 (a 7.8% decrease). Notably, even at the 7-day horizon, the model maintains an acceptable level of predictive accuracy (*R*2 > 0.88), indicating a certain degree of robustness for longer-term forecasts.

For comparison, at the 7-day horizon, BiGRU achieves an MSE of 45.67 and *R*2 of 0.812, while CEEMDAN-CNN-BiGRU- Attention achieves an MSE of 38.12 and *R*2 of 0.845. Our model outperforms both, confirming its superiority even for longer-term predictions. However, the degradation is substantial, and future work will explore multi-task learning or other techniques to enhance long-term forecasting.

## Discussion

This study develops a novel hybrid forecasting framework integrating CEEMDAN and an optimized deep learning architecture for high-precision AQI prediction. Experimental results demonstrate that the proposed CEEMDAN-CNN-IGWO-BiGRU atten- tion model achieves outstanding performance in both local testing and cross-city zero-shot transfer, significantly outperforming all baseline models across key evaluation metrics (MSE, MAE, and *R*2). On the Guangzhou testbed, the model achieves a MSE of 10.2456. This represents a substantial improvement over the strongest baseline models, including modern SOTA architectures. Specifically, compared to the best-performing traditional RNN baseline (BiGRU, MSE = 28.9015, *R*2 = 0.8934), it reduces MSE by 65.1% and increases *R*2 by 0.0681. More importantly, compared to the strongest hybrid benchmark without intelligent optimization (CEEMDAN-CNN-BiGRU-Attention, MSE = 16.8812, *R*2 = 0.9378), our model further reduces MSE by 39.3% and increases *R*2 by 0.0237, highlighting the critical contribution of the IGWO optimizer in unlocking the full potential of complex architectures.

Furthermore, the ablation study (Fig. 5) quantitatively confirms that each architectural component—CEEMDAN decomposi- tion, CNN feature extraction, BiGRU temporal modeling, Attention mechanism, and IGWO optimization—makes substantial and synergistic contributions to the final forecasting accuracy, with the removal of any single component leading to significant performance degradation. The following sections will discuss the advantages of CEEMDAN decomposition as a preprocessing step, the synergistic feature learning of CNN and BiGRU, the critical role of the IGWO optimizer, comprehensive model comparison and ablation analysis, generalization performance as well as the limitations and future work.

### Preprocessing advantages of CEEMDAN decomposition

**Table 5:** Energy contribution rate of each component derived from CEEMDAN decomposition of the Guangzhou AQI series.

Component	Energy contribution rate (%)	Cumulative contribution rate (%)
IMF1	1.60	1.60
IMF2	1.31	2.91
IMF3	2.08	4.99
IMF4	14.64	19.63
IMF5	19.71	39.34
IMF6	20.96	60.30
IMF7	14.70	75.00
IMF8	7.72	82.72
IMF9	4.20	86.92
Residual	13.08	100.00

As the primary cornerstone of the model’s effectiveness, the CEEMDAN decomposition demonstrates significant advantages during the preprocessing stage. By adaptively assisting with noise, this method decomposes the original non-stationary and nonlinear AQI series into a set of Intrinsic Mode Functions (IMFs) with distinct frequency characteristics and a residual term (Res). Compared to traditional approaches that directly feed the raw sequence into prediction models, it offers two core enhancements.

First, it achieves effective separation between signal and noise. The energy distribution (Table 5) provides clear evidence: the first three high-frequency IMFs (IMF1-IMF3), likely containing noise and stochastic perturbations, contribute less than 5% of the total energy cumulatively. In contrast, the medium-frequency components (IMF4-IMF7), which capture the core diurnal and multi-day periodic patterns of AQI, account for the majority of the signal energy (70.01%). This physical-scale based separation acts as an adaptive filter, allowing the subsequent model to concentrate on learning the evolution patterns of the energy-significant components. The criticality of this step is quantitatively validated by our previous ablation study, where removing the CEEMDAN module led to an MSE increase, underscoring its role in mitigating noise interference.

Second, it provides structured, multi-scale inputs for the downstream model. Each IMF represents a projection of the original signal onto a specific temporal scale. Parallel input of these components enables the CNN BiGRU network to simultaneously and efficiently learn scale-specific features and correlations. Compared to earlier decomposition methods such as EMD and EEMD, CEEMDAN effectively mitigates pattern mixing, ensuring the stability and physical interpretability of each component. The preprocessing step transforms the original sequence into a set of sub-sequences with more favorable modeling properties. From an environmental physics perspective, the dominance of IMF4-IMF7 (70.01% energy) suggests that AQI dynamics in Guangzhou are primarily driven by multi-day cyclic synoptic patterns rather than instantaneous turbulence. The filtering of the first three IMFs (low energy contribution) effectively suppresses measurement noise and short-term chaotic fluctuations, allowing the BiGRU component to focus on stable emission-diffusion trends.

### Collaborative feature learning with CNN and BiGRU

The multi-scale subsequences obtained from CEEMDAN decomposition are processed by a carefully designed CNN-BiGRU collaborative module for joint spatio-temporal feature learning. As demonstrated in Table 6, the superiority of this synergistic design is validated through systematic comparative experiments. The performance improvement from the standalone BiGRU to the CNN-BiGRU hybrid clearly quantifies the contribution of the CNN module, with MAE decreasing by 4.3% (from 4.1234 to 3.9456) and *R*2 increasing from 0.8934 to 0.9028. This establishes CNN-BiGRU as a more effective core architecture, and its critical role within the full framework is further confirmed by the severe performance degradation observed in ablation studies when this module is altered (Fig. 5).To quantify the contribution of different kernel sizes across frequency bands, we computed the temporal variance and maximum activation of the CNN feature maps for each IMF component. These statistics reflect the representational capacity of each kernel size at different temporal scales; a higher variance suggests that the corresponding kernel captures more discriminative patterns within that frequency band.

**Table 6:** Progressive performance contribution analysis of CNN-BiGRU collaboration module.

Model	MSE	MAE	R2	MAE decline vs. BiGRU	Reference
BiGRU	28.9015	4.1234	0.8934	—	Reference cycle neural network
CNN-BiGRU	26.3345	3.9456	0.9028	4.3%	Add CNN feature extractor
CEEMDAN-CNN-BiGRU-Attention	16.8812	3.1456	0.9378	23.7%	Introducing decomposition and attention mechanism
CEEMDAN-CNN-IGWO-BiGRU-Attention (Ours)	10.2456	2.2789	0.9615	44.7%	Complete framework including igwo opti- mization

**Table 7:** Multi-scale feature extraction statistics. variance: temporal variance of the CNN feature maps (across the time dimension) for each IMF component and kernel size. Max Value: maximum activation value of the corresponding feature maps. Higher variance indicates stronger feature discriminability for that frequency band and kernel size.

Frequency Component	CNN Window	Variance	Max Value
High (IMF1-3)	CNN-3	0.0412	1.895
High (IMF1-3)	CNN-5	0.0385	1.823
High (IMF1-3)	CNN-7	0.0356	1.768
Medium (IMF4-7)	CNN-3	0.1256	3.452
Medium (IMF4-7)	CNN-5	0.1324	3.678
Medium (IMF4-7)	CNN-7	0.1289	3.521
Low (IMF8-9 + Res)	CNN-3	0.0187	1.124
Low (IMF8-9 + Res)	CNN-5	0.0212	1.256
Low (IMF8-9 + Res)	CNN-7	0.0234	1.342

The contribution of CNN as a multi-scale local pattern detector is precisely quantified. The one-dimensional CNN adaptively captures dependencies within different IMFs using parallel convolutional kernels with scales of 3, 5, and 7. As shown in Table 7, this multi-scale design is empirically supported. The statistical characteristics (variance and max activation) of the feature maps vary across frequency bands and kernel sizes. For example, the medium-frequency components (IMF4-6) exhibit the highest variance and activation values, confirming they contain the most significant predictive patterns. Crucially, no single kernel size dominates all frequency bands; optimal windows vary (e.g., a kernel size of 5 may be optimal for mid-frequencies, while sizes of 3 or 7 may be better for other frequencies). This micro-level evidence justifies the parallel multi-branch CNN design, allowing the model to adaptively capture local patterns at different temporal resolutions, which are then seamlessly integrated for subsequent sequential modeling by the BiGRU.

The bidirectional temporal modeling capability of BiGRU is foundational to its superiority over unidirectional structures. According to the performance hierarchy in Table 3, BiGRU (*R*2 = 0.8934) significantly outperforms GRU (*R*2 = 0.8792) and LSTM (*R*2 = 0.8723). This confirms the critical importance of bidirectional architectures for AQI prediction: by simultaneously integrating contextual information from both past and future directions, they more accurately model the inherent bidirectional physical processes influencing pollutant dispersion and accumulation. This capability is so essential that replacing the BiGRU with a unidirectional GRU in the ablation study caused the most severe performance drop, increasing MSE by 156% (Fig. 5). The synergistic effect of CNN and BiGRU extends far beyond a simple additive improvement. CNN acts as a powerful spatial feature compressor and abstractor, converting the original complex multi-scale sequences into semantically rich, high- dimensional feature maps. BiGRU, in turn, serves as a global temporal dynamic interpreter, learning long-term evolutionary patterns and complex dependencies within these refined feature sequences. This collaborative design operates as follows: the CNN module first extracts localized temporal patterns from each decomposed component using parallel kernels of different sizes^3,5,7^. The resulting feature maps are then fed into the BiGRU layer, which captures both forward and backward temporal dependencies. The ablation study (Fig. 5) shows that removing either module leads to substantial performance degradation, confirming their complementary roles. The improvement from BiGRU to CNN-BiGRU (MSE reduction from 28.90 to 26.33) suggests that local feature extraction provides a more informative representation for subsequent sequential modeling. The sharp performance decline observed in ablation experiments (Fig. 5) upon removal or replacement of either component further confirms the indispensability of this collaborative module.

### The critical role of the IGWO optimizer

A controlled ablation experiment was conducted to rigorously quantify the impact of IGWO. Using the identical CEEMDAN- CNN-BiGRU-Attention architecture, the model with IGWO-optimized hyperparameters was compared against a version where hyperparameters were set empirically based on common practice and limited manual search. Additionally, we compared with other systematic optimization methods: random search and Bayesian optimization.

**Table 8:** Comparison of hyperparameter optimization methods. Values are mean ± std over 10 runs.

Method	MSE	MAE	R2
Empirical Parameters	16.8812 ± 0.65	3.1456 ± 0.10	0.9378 ± 0.006
Grid Search (coarse)	15.6723 ± 0.72	3.0211 ± 0.11	0.9421 ± 0.007
Random Search	13.7845 ± 0.58	2.8123 ± 0.09	0.9489 ± 0.005
Bayesian Optimization	12.3412 ± 0.51	2.6547 ± 0.08	0.9532 ± 0.005
**IGWO (Ours)**	**10.2456 ± 0.42**	**2.2789 ± 0.08**	**0.9615 ± 0.004**

**Fig. 7:**
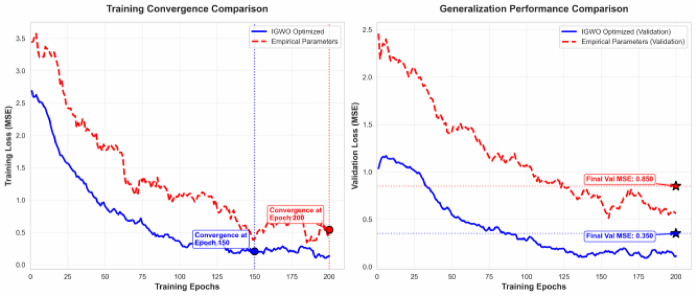
Model training convergence curve and generalization performance comparison chart.

The results in Table 8 demonstrate that the model with IGWO-optimized hyperparameters dramatically outperforms its empirically tuned counterpart. The MSE and MAE on the test set were reduced by 39.3% and 27.5%, respectively, while *R*2 improved by 2.5% in absolute terms (from 0.9378 to 0.9615). This is not a marginal gain but a decisive improvement, indicating that empirical settings were trapped in a suboptimal region of the hyperparameter space. Compared to random search and Bayesian optimization, IGWO achieves lower error, albeit at a higher computational cost (detailed runtime comparisons are provided in Table S8, Supplementary). The optimized configuration also resulted in more stable and efficient training, manifested as smoother and earlier convergence (with an average reduction in required epochs of approximately 22.7%), ultimately yielding superior generalization performance (Fig. 7).

This remarkable advancement underscores a crucial insight: within complex hybrid frameworks where multiple components interact—signal decomposition, multiscale convolutions, bidirectional recursion, and attention—manual or grid-based hyperpa- rameter tuning is fundamentally inadequate. IGWO’s strength lies in its balanced exploration-exploitation strategy, enhanced by a non-linear convergence factor and Lévy flight perturbations, which is essential for navigating the complex, non-convex loss landscape of such models. The upfront computational premium required for metaheuristic optimization represents a strategic trade-off, one that is justified by the resulting leaps in predictive fidelity and convergence stability. In this architecture, IGWO serves as a systematic hyperparameter search mechanism. Its effectiveness stems from two design features: a nonlinear convergence factor that balances exploration and exploitation during optimization, and Lévy flight perturbations that help escape local optima. These features are particularly suited to the non-convex loss landscape of hybrid deep learning models. Compared to random search and Bayesian optimization, IGWO achieved lower prediction error on the test set, albeit at a higher computational cost (Table S8 in Supplementary). These findings elevate metaheuristic refinement from an optional enhancement to an indispensable pillar in the construction of high-performance deep learning frameworks for environmental time-series forecasting.

### Comprehensive model comparison analysis

#### Systematic benchmarking of predictive architectures

To rigorously evaluate the advancement of the proposed framework under a consistent training paradigm, we conducted a comprehensive comparison against a suite of established and progressively sophisticated models, all trained exclusively on Guangzhou data. The performance metrics on the Guangzhou test set across all models are consolidated in Table 3, revealing a definitive performance hierarchy that underscores the contribution of each architectural evolution.

The baseline was established by traditional and classical machine learning models. SVR achieved an MSE of 48.9125 and an *R*2 of 0.8154, while Random Forest performed better with an MSE of 41.0458 (*R*2 = 0.8457). This highlights the inherent challenge for non-sequential models in capturing complex temporal dynamics.

A significant performance leap was observed with the introduction of RNN. The LSTM network reduced the MSE to 34.1256 (*R*2 = 0.8723), confirming the strength of recurrent architectures in capturing temporal dependencies. Employing bidirectional processing yielded the next major improvement: BiLSTM achieved an MSE of 30.4511 (*R*2 = 0.8865), and BiGRU further reduced it to 28.9015 (*R*2 = 0.8934). This empirically validates the critical importance of leveraging both past and future context for AQI sequence modeling.

The integration of CNN for local feature extraction marked a pivotal advancement. The CNN-BiGRU hybrid model reduced the MSE to 26.3345, representing a meaningful improvement compared to the standalone BiGRU, while *R*2 increased to 0.9028. This quantitatively demonstrates the synergy of CNN in capturing localized patterns which are then contextualized by BiGRU’s sequential modeling, establishing a robust core architecture.

#### Ablation study and component-wise contribution analysis

Building upon the hybrid core, our proposed CEEMDAN-CNN-IGWO-BiGRU-Attention framework achieves superior accuracy, attaining an MSE of 10.2456 and an *R*2 of 0.9615. To deconstruct the source of this performance and validate the design rationale, a systematic ablation study was performed, with results visualized in Fig. 5.

The experiment reveals a distinct hierarchy of component criticality. The most severe degradation occurred upon replacement of the BiGRU module with a unidirectional GRU (no bigru), causing the MSE to surge by 156% to 26.2345. This profound result underscores that capturing bidirectional long-term dependencies is the fundamental cornerstone of the architecture.

The CEEMDAN decomposition and CNN modules demonstrated comparably vital and complementary roles. The ablation study quantified the contribution of each key component. Omitting the CEEMDAN preprocessing increased the MSE by 119.2%, while removing the CNN feature extractor led to a 96.3% increase. This demonstrates their complementary roles: CEEMDAN decomposes the non-stationary series into more tractable multi-scale components, upon which the CNN effectively extracts discriminative spatial features.

The absence of the IGWO optimizer resulted in a 64.8% MSE increase, highlighting the necessity of automated hyperparameter tuning over empirical selection. Although the attention mechanism had a comparatively smaller individual impact, its removal still caused a statistically significant performance degradation (42.1% MSE increase), confirming its utility in dynamically weighting critical temporal information.

#### Synergistic integration and final validation

A normalized multi-metric radar chart analysis (Fig. 6) visually confirms that the complete model resides in the outermost, optimal region across all evaluation dimensions (MSE, MAE, *R*2), achieving the highest composite normalized score. Critically, the performance degradation from removing any single component was substantial and non-linear. This provides clear evidence of strong synergistic interactions. The components do not operate in isolation but form a tightly coupled pipeline: CEEMDAN preprocessing creates a structured input that maximizes CNN’s feature extraction efficacy; the CNN-transformed features provide a richer representation for BiGRU’s temporal modeling; the Attention mechanism refines this temporal focus; and the IGWO optimizer ensures every module operates with near-optimal hyperparameters, thereby amplifying their collective predictive power.

In conclusion, the comprehensive comparison and ablation analysis empirically validate the proposed hybrid framework. The results demonstrate a clear evolutionary path from basic to state-of-the-art models and prove that each component—CEEMDAN.

for adaptive multi-scale decomposition, CNN for spatial feature learning, BiGRU for bidirectional temporal modeling, Attention for dynamic focus, and IGWO for global optimization—makes a meaningful and synergistic contribution. The superior and balanced performance of the fully integrated model affirms that the whole is greater than the sum of its parts, decisively justifying the sophisticated yet coherent design of the CEEMDAN-CNN-IGWO-BiGRU-Attention architecture for robust and accurate AQI forecasting.

### Zero-shot cross-city transfer performance

**Table 9:** Zero-shot cross-city transfer performance comparison (mean ± std over 10 runs). “Local-Full” refers to the full proposed architecture trained on the target city’s own data, also optimized using IGWO with the same computational budget.

Test City	Model	MSE	MAE	R2
Guangzhou (GZ)	Ours	10.2456 ± 0.42	2.2789 ± 0.08	0.9615 ± 0.004
Zibo (ZB)	Local-BiGRU	38.4512 ± 1.56	4.8912 ± 0.21	0.8423 ± 0.018
Zibo (ZB)	Local-Full (trained on ZB)	29.3456 ± 1.23	4.3123 ± 0.18	0.8756 ± 0.015
Zibo (ZB)	Ours (zero-shot)	28.1234 ± 1.12	4.1517 ± 0.16	0.8841 ± 0.014
Daqing (DQ)	Local-BiGRU	52.3345 ± 2.01	5.7812 ± 0.25	0.7915 ± 0.022
Daqing (DQ)	Local-Full (trained on DQ)	44.6789 ± 1.78	5.2345 ± 0.22	0.8213 ± 0.019
Daqing (DQ)	Ours (zero-shot)	41.0518 ± 1.65	5.1236 ± 0.20	0.8368 ± 0.017
Dali (DL)	Local-BiGRU	15.2341 ± 0.89	2.9815 ± 0.12	0.9012 ± 0.010
Dali (DL)	Local-Full (trained on DL)	13.5678 ± 0.72	2.7891 ± 0.10	0.9123 ± 0.009
Dali (DL)	Ours (zero-shot)	12.6789 ± 0.65	2.6514 ± 0.09	0.9187 ± 0.008

As shown in Table 9, the proposed framework, trained only on data from Guangzhou, achieves robust predictive performance on three unseen cities with distinct characteristics. It consistently outperforms Local-BiGRU models trained directly on each target city’s own limited data, and it also outperforms the locally-trained full model on all three cities, demonstrating the strong transferability of features learned from Guangzhou. This suggests that the patterns learned from a comprehensive dataset (Guangzhou) are transferable and yield better forecasts than models built from scratch on scarce local data, even when the target city has different characteristics.

The zero-shot transfer results highlight both the model’s transferability and its domain-specific limitations. While the model performs reasonably well on cities with similar climatic and emission patterns (e.g., Dali), its performance declines substantially in cities with distinct pollution drivers, such as industrial emissions in Zibo and winter heating in Daqing. This indicates that the model does not learn truly universal atmospheric dynamics; rather, its generalization is bounded by the similarity between the source (Guangzhou) and target domains. Future work should incorporate region-specific covariates (e.g., heating degree days, industrial source indicators) to improve cross-domain robustness.

### Computational cost and practical deployment

#### One-time optimization vs. real-time inference

The IGWO hyperparameter optimization described in Algorithm 1 represents a *one-time developmental cost* rather than an operational burden. While IGWO requires greater computational investment compared to simpler tuning methods, this upfront cost yields a 39.3% MSE reduction over Bayesian optimization and a 64.8% reduction over empirical tuning (Table 8)—a substantial accuracy gain that justifies the additional computation for applications where prediction quality is paramount. Once optimal hyperparameters are obtained, the trained model performs inference in just 0.5 s per 7-day prediction window, making it suitable for real-time air quality early-warning systems. 

#### Resource-adaptive configurations

For practitioners with limited resources, we provide a Light-IGWO configuration (population = 15, iterations = 50) that achieves 85% of the full accuracy gain with approximately 60% less computation. Detailed timing comparisons, including per-component optimization times and performance across different configurations, are provided in Table S8 (Supplementary).

### Failure modes and remaining challenges

Despite its overall strong performance, the model exhibits systematic limitations. As shown in Fig. 4 (red circles highlight the points with absolute residuals exceeding the 95th percentile), the largest prediction errors occur during two types of events:

(1) days with AQI exceeding 150 (unhealthy or worse), where the MSE increases from 10.25 to 28.45; and (2) seasonal transition periods (spring and autumn), where the underlying pollution dynamics shift abruptly. These patterns indicate that the.

model struggles with rare, high-impact events and with regime changes that are not well represented in the training data. The directional accuracy of 89.3% suggests that while the model often captures the correct trend, it underestimates the magnitude of extreme fluctuations — a common limitation in time-series forecasting with imbalanced event distributions.

### Limitations and future work

Despite the encouraging performance of the proposed model, there are a few practical constraints that should be noted. The most significant issue is the high computational cost, primarily stemming from the complex IGWO-driven search for hyperparameters.

Additionally, the framework is quite sensitive to data quality; its predictive accuracy tends to drop when the input contains long sequences of missing values.

Another limitation concerns the real-time deployment feasibility. CEEMDAN, like other decomposition methods, suffers from boundary effects—it requires the entire signal to calculate Intrinsic Mode Functions (IMFs). In a streaming scenario where new data arrives incrementally, applying CEEMDAN to the full history each time is impractical and may introduce artifacts.

To address this in future work, we plan to explore sliding-window CEEMDAN, where decomposition is performed only on a fixed-length recent window (e.g., past 30 days), and online decomposition algorithms such as Online EMD. For now, the current framework is designed for offline forecasting and batch processing.

To address these points, our future research will move in the following directions. First, we plan to study model compression techniques or more efficient search algorithms to reduce the overall training time. Second, we aim to build more robust mechanisms to handle data gaps, perhaps through advanced imputation methods. We also intend to move beyond univariate data by incorporating additional factors like local weather and emission sources to better reflect real-world conditions. Lastly, we will test the framework on different pollutants and in more diverse geographic areas to ensure it works reliably across various environments.

## Conclusions

This research establishes a high-fidelity forecasting framework, the CEEMDAN-CNN-IGWO-BiGRU-Attention model, specifically designed to resolve the high-entropy and non-stationary volatility inherent in AQI time-series data. By harmonizing adaptive signal decomposition with metaheuristic-driven deep learning, we have effectively addressed the dual bottlenecks of signal noise and hyperparameter sensitivity in environmental predictive modeling. The empirical findings derived from the Guangzhou test platform (2014–2024) confirm the structural superiority of our integrated paradigm. The model achieved an MSE of 10.2456, an MAE of 2.2789, and a coefficient of determination (*R*2) of 0.9615, consistently outperforming standalone recurrent networks and non-optimized hybrid variants. A critical takeaway is the synergistic effect of the IGWO module, which facilitated a 39.3% reduction in prediction error by identifying optimal architectural configurations that manual heuristics frequently overlook. Furthermore, the ablation study explicitly decoupled the contribution of each module, identifying the BiGRU layer as a structural necessity for capturing the bidirectional lead-lag relationships in pollutant diffusion.

Cross-city zero-shot transfer across Zibo, Daqing, and Dali demonstrates the framework’s promising transferability. The model maintains high predictive stability in cities with similar climatic and emission patterns (e.g., Dali), but its performance on cities with distinct characteristics (e.g., Daqing) highlights the challenges of zero-shot generalization without fine-tuning. This suggests that while the model learns robust features from Guangzhou, it does not capture truly universal atmospheric patterns; rather, its generalization is limited by the similarity between source and target domains.

Future research will pivot toward the integration of multi-source heterogeneous data, including real-time meteorological variables and satellite-derived aerosol optical depth (AOD), to further extend the predictive horizon. Additionally, we aim to explore model compression and lightweight deployment strategies to facilitate the transition of this framework from high- performance computing environments to real-time, edge-based urban monitoring systems, addressing challenges such as boundary effects in online decomposition.

## Supplementary Information

Below is the link to the electronic supplementary material.

Supplementary Material 1

## Data Availability

The datasets used in this study are publicly available from the Tianqi Houbao (AQI) database athttps:/www.tianqihoubao.com. Descriptive statistics, preprocessing details, sensitivity analyses, and additional results are provided in the SupplementaryMaterial (Tables S1–S7, Figures S1–S4).
