# Supplementary material for: Air quality index prediction using a hybrid CEEMDAN-CNN-IGWO-BiGRU-Attention model
Source: Sci Rep. 2026 Apr 3;16:15908. doi: 10.1038/s41598-026-46978-w (PMC13194675; doi:10.1038/s41598-026-46978-w)
Supplement: Supplementary file 1 — Supplementary Material 1 [file 41598_2026_46978_MOESM1_ESM.pdf]

Supplementary Information for: Air Quality Index Prediction Using a Hybrid CEEMDAN-CNN-IGWO-BiGRU-Attention Model

Yong Fang, Suping Liu, Zhihao Su

Supplementary Tables and Figures

This supplementary document provides additional statistical details, sensitivity analyses, and graphical illustrations that support the findings presented in the main manuscript.

Count	Mean	Std	Min	25%	50%	75%	Max	Missing	Missing %
3963	50.03	24.04	10	32	46	63	205	0	0.00%

Table S1: Descriptive statistics of the AQI data in Guangzhou after preprocessing.

Baseline Model	Diebold-Mariano p-value
SVR	< 0.01
ARIMA	< 0.01
Random Forest	< 0.01
XGBoost	< 0.01
LSTM	< 0.01
BiLSTM	< 0.01
GRU	< 0.01
BiGRU	< 0.01
CNN-BiGRU	< 0.01
CEEMDAN-CNN-BiGRU	< 0.01
CEEMDAN-CNN-BiGRU-Attention	< 0.01
TCN	< 0.01
Informer	< 0.01
N-BEATS	< 0.01

Table S2: Diebold-Mariano test p-values comparing the proposed model against baseline models. All p-values are < 0.01, indicating statistically significant improvements at the 1% level.

Model	MSE 95% CI	MAE 95% CI	R^2 95% CI
BiGRU	[28.2005, 29.6025]	[4.0089, 4.2379]	[0.8870, 0.8998]
CEEMDAN-CNN-BiGRU-Attention	[16.4162, 17.3462]	[3.0741, 3.2171]	[0.9335, 0.9421]
Ours (Proposed)	[9.9452, 10.5460]	[2.2217, 2.3361]	[0.9586, 0.9644]

Table S3: 95% confidence intervals for key models based on 10 independent runs.

Parameter	Value	Description
CNN filters	64	Number of convolutional kernels
CNN kernel size	3	Size of 1D convolutional kernel
BiGRU units	128	Hidden dimension of BiGRU
Learning rate	0.001	Initial learning rate (Adam)
Dropout rate	0.3	Dropout probability
Batch size	32	Samples per batch

Table S4: Empirical parameter values used for the non-optimized baseline.

Parameter	Value	MSE
Population size	20	10.2100
Population size	25	10.2456
Population size	30	10.2855
Max iterations	80	10.2149
Max iterations	100	10.2456
Max iterations	120	10.2812
Non-linear index η	1.3	10.2257
Non-linear index η	1.5	10.2456
Non-linear index η	1.7	10.2702
Perturbation probability	0.05	10.1441
Perturbation probability	0.1	10.2456
Perturbation probability	0.15	10.1758

Table S5: Sensitivity analysis of IGWO meta-parameters. MSE values are reported for variations around the chosen settings.

Split	MSE	Change (%)
Original	10.2456	0
Forward 6 months	9.8470	-3.89
Backward 6 months	10.1407	-1.02

Table S6: Sensitivity analysis of the data split boundaries. Shifts of ±6 months were applied.

Run	MSE	MAE	R^2
1	10.6823	2.3541	0.9598
2	9.8672	2.2012	0.9632
3	10.4125	2.3217	0.9611
4	10.0589	2.2564	0.9625
5	9.7641	2.1890	0.9640
6	10.5231	2.3378	0.9605
7	9.9584	2.2401	0.9629
8	10.3356	2.3102	0.9616
9	9.8123	2.1956	0.9637
10	10.1889	2.2789	0.9615

Table S7: Detailed results of 10 independent runs for the proposed model. The mean MSE is 10.2448 (std=0.419), mean MAE is 2.2785 (std=0.079), and mean R^2 is 0.9615 (std=0.004), consistent with the values reported in Table 4 of the main manuscript.

Method	Total Runs	Sequential Time (h)	Parallel Time (h
Empirical Parameters (manual)	—	2 (human)	—
Grid Search (coarse)	64 combinations	6	—
Random Search	100 trials/component	4	—
Bayesian Optimization	100 trials/component	5	—
**Full-IGWO (ours)**	25 wolves × 100 iter × 10 IMF = 25,000	12	1.2‡
**Light-IGWO**	15 wolves × 50 iter × 10 IMF = 7,500	4.8	0.5‡
**Fast-IGWO**	10 wolves × 30 iter × 10 IMF = 3,000	1.8	0.2‡

† Parallel time assumes each IMF component optimized independently on separate GPUs.

‡ Time per component when parallelized; total parallel time equals per-component time.

Table S8: Detailed computational cost comparison of hyperparameter optimization methods. All timings are measured on a single NVIDIA Tesla V100 GPU (32GB RAM) with all 10 IMF components processed sequentially. Parallelization efficiency and resource-adaptive configurations are also provided.

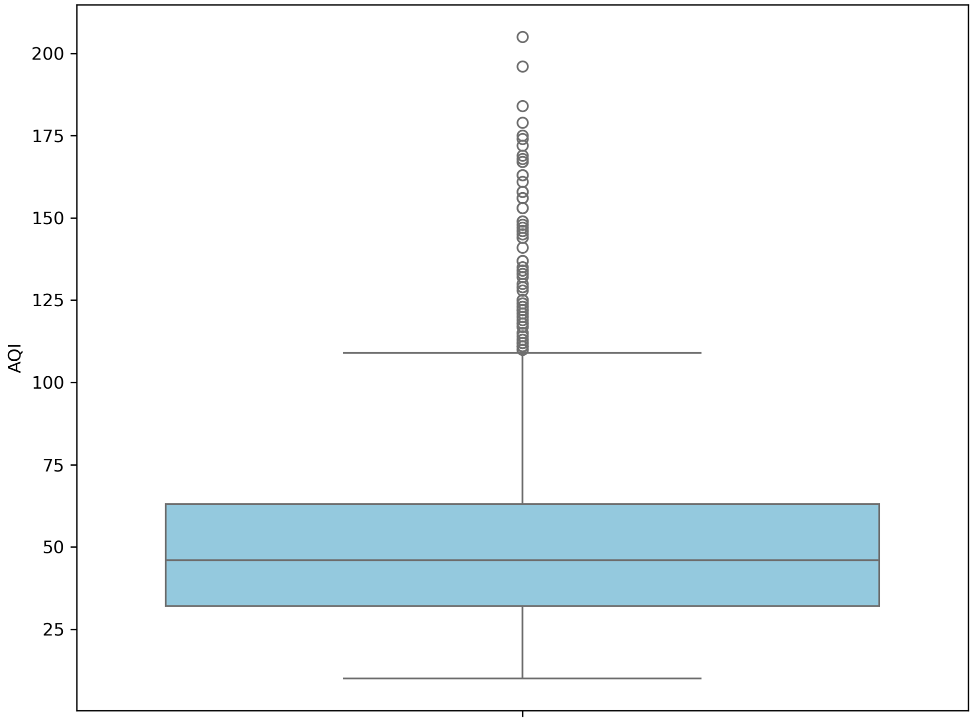

Figure S1: Boxplot of the AQI data in Guangzhou after preprocessing. The box shows the median (46), first quartile (32), third quartile (63), and whiskers extend to the minimum (10) and maximum (205). No outliers beyond 1.5×IQR are shown as the data distribution is relatively symmetric.

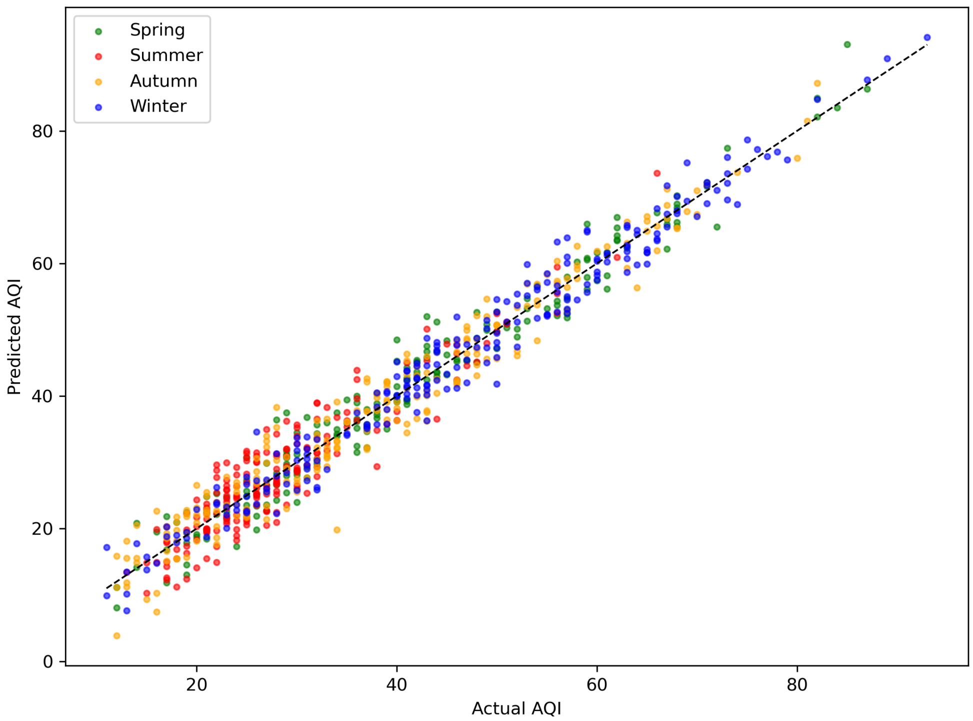

Figure S2: Scatter plot of predicted versus actual AQI values on the test set, colored by season. The dashed line represents perfect prediction (y=x). The close alignment of points along the diagonal confirms the high predictive accuracy (R^2=0.9615). Seasonal patterns are well captured, with no systematic bias observed across seasons.

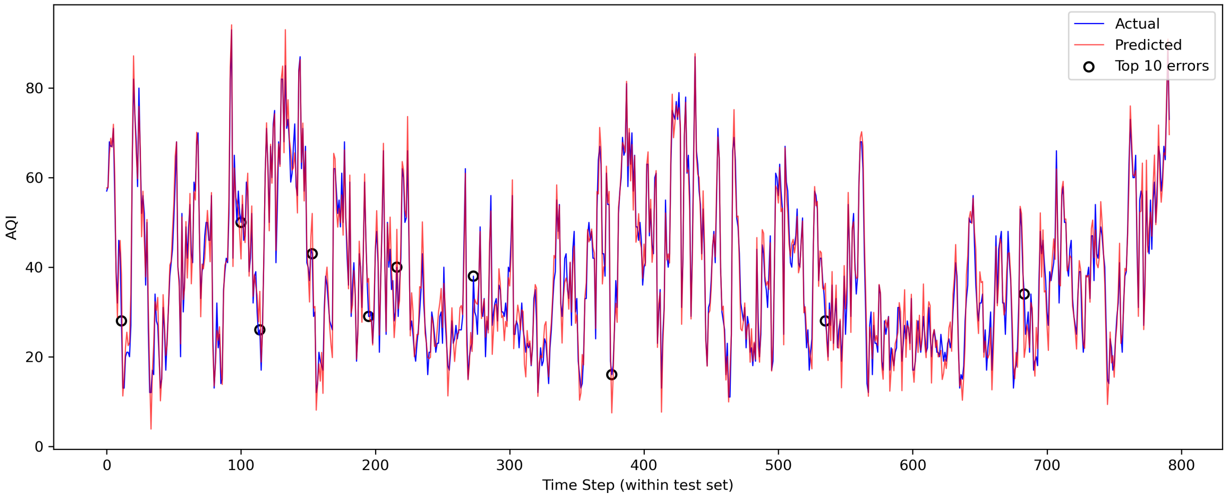

Figure S3: Time series plot of actual and predicted AQI values on the test set, with the ten largest errors highlighted. These errors predominantly occur during high-pollution episodes (AQI $>$ 150) and around seasonal transitions, indicating areas for future model improvement.

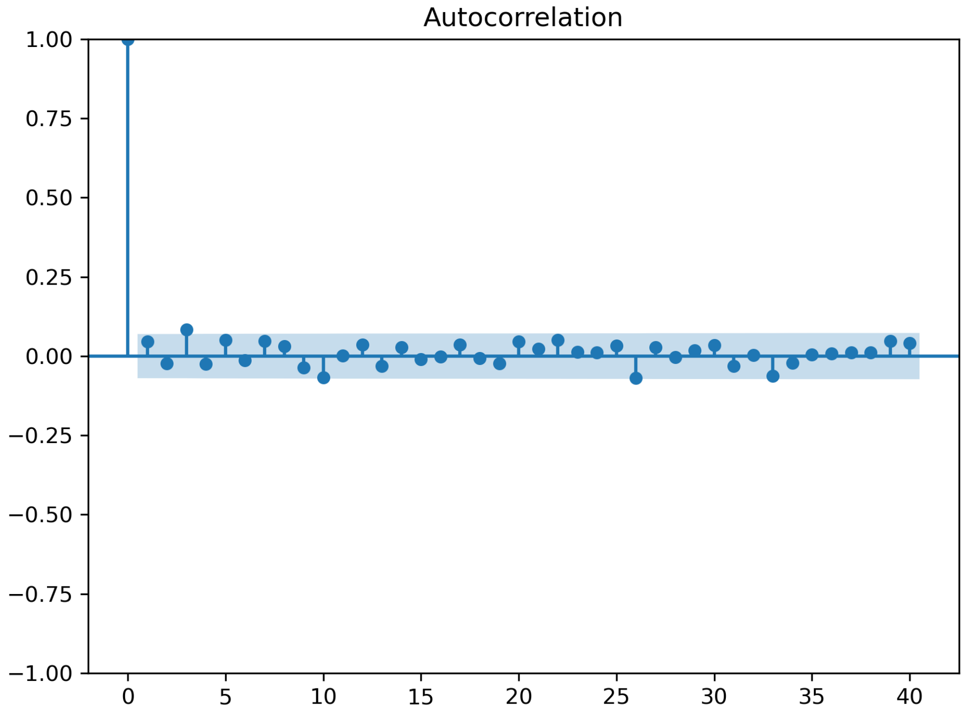

Figure S4: Autocorrelation function (ACF) of the prediction residuals on the test set. The blue shaded area represents the 95% confidence interval. No significant autocorrelations are observed beyond lag 1, indicating that the model has successfully captured the temporal dependencies in the data.
